# The Effects of Instruction on the Frequency and Characteristics of Involuntary Autobiographical Memories

**DOI:** 10.1371/journal.pone.0157121

**Published:** 2016-06-13

**Authors:** Krystian Barzykowski, Agnieszka Niedźwieńska

**Affiliations:** Applied Memory Research Laboratory, Institute of Psychology, Jagiellonian University, Krakow, Poland; University of L'Aquila, ITALY

## Abstract

The present study investigated the effects of experimental instruction on the retrieval of involuntary autobiographical memories (IAMs). In previous studies of IAMs, participants were either instructed to record only memories (henceforth, the restricted group) or any thoughts (henceforth, the unrestricted group). However, it is unknown whether these two different types of instructions influence the retrieval of IAMs. The most recent study by Vannucci and her colleagues directly addressed this question and demonstrated that the frequency and phenomenological characteristics of IAMs strongly depended on the type of instruction received. The goal of the present study was to replicate these results while addressing some limitations of the Vannucci et al. study and to test three possible mechanisms proposed to explain the effect of instructions on the retrieval of IAMs. Our results accord well with the data presented by Vannucci et al. When participants were instructed to record only IAMs (the restricted group), they reported more memories and rated them as being retrieved in a more goal-oriented fashion. Their memories also were less clear, vivid, detailed and were less frequently accompanied by physiological reactions, compared to memories reported by the participants in the unrestricted group. In addition, the events to which the memories referred were rated as more unusual and personal by the restricted group. These results are consistent with the assumption that retrieval of IAMs depends on the type of instructions used in a study. In addition, our results suggest that one of the main mechanisms underlying the higher frequency of IAMs in the restricted group may be participants’ ability to monitor the stream of consciousness and to extract autobiographical content from this flow. Further implications of the effect of instructions for IAMs research are discussed.

## Introduction

Every time we try to recall something from our personal past (e.g. the last time I had a headache) we use our autobiographical memory. However, sometimes memories can suddenly pop into mind, without a preceding attempt to recall anything. The latter memories, called involuntary autobiographical memories (IAMs), come to mind automatically without any conscious attempt to retrieve them [1, 2], whereas the former memories, called voluntary memories, result from an intentional attempt to retrieve them from memory, which typically involves an effortful search [3–6]. Recent research by Barzykowski and Staugaard [7] highlights the importance of describing IAMs in terms of their lack of cognitive effort (e.g. [8]) and the lack of intention to retrieve them.

There has been growing interest in IAMs over last two decades. The involuntary recall of one’s personal past may have a significant effect on a person’s mood and well-being (e.g. [9]. IAMs may be important in relation to various mental disorders, e.g. depression [10, 11] and to mental processes, such as identity, by providing a sense of continuity across time [12]. Involuntary remembering also may be important in relation to both memory impairment (e.g. amnestic syndrome) and unusually good autobiographical memory [13], both of which may be burdensome. The first case is related to the inability to voluntarily recall memories from one’s personal past [14]. However, information may often be successfully retrieved in an involuntary and automatic fashion [14]. The second case is related to an ability to recall vast amounts of personally irrelevant information without the use of mnemonics [13, 15]. This phenomenon can be especially problematic when individuals are dominated by constant, unstoppable, and uncontrollable memories of their personal past. Research on IAMs has important implications for our understanding of intrusive memories in PTSD and related disorders [16]. For example, studies on the cognitive mechanisms of IAMs may be important for developing strategies to cope with intrusive memories.

Developing knowledge about IAMs and the mechanisms that underlie them is an important step towards gaining insight into the nature and functioning of memory processes and human cognition, in general. Therefore, the empirical examination of IAMs under well-controlled experimental conditions may contribute to both everyday life and future research advances. An experimental, laboratory method of investigating IAMs has been developed [17, 18, 19] that enables controlled investigation and can stimulate advances in this research domain. All methodological issues related to this procedure should be carefully considered to help develop the best possible final procedural format. Our study served this general purpose.

There are three main research strategies used in studies of IAMs (see [20], for a more detailed review): survey methods (e.g. [21–22]), structured diaries [1, 23–26], and experimental, laboratory methods [18–19, 27–29]. Most studies, so far, have instructed participants to record all the IAMs they experienced during a specified period of time (e.g. [1, 17, 19, 23–27]). However, some studies have instructed participants to record any involuntary thoughts that popped into their mind; IAMs were then selected from the pool of recorded entries (e.g. [18, 30]. As the first procedure restricts the set of participants’ responses only to IAMs, compared with the second procedure, henceforth, we will call these two conditions the restricted group and the unrestricted group, respectively.

Mace [31] highlights the fact that a prior intention to retrieve memories lies at the heart of the involuntary vs. voluntary distinction. Intentional retrieval corresponds to what has been termed ‘retrieval mode’ [32], in which ‘the cognitive system is prepared for or expects memory construction and recollection’ ([33], p. 1379). Retrieval mode is thought to involve the activation of schematic and strategic retrieval processes, such as emotional regulation. Retrieval cues and episodic memories are processed differently in this cognitive state than they are under retrieval instructions when individuals do not intentionally recall the past (e.g. [34–35]). Therefore, it can be reasonably argued that the retrieval of IAMs may be influenced by whether or not study participants are informed that they are to report memories [29]. This problem is related to a broader methodological question; namely, the extent to which experimental procedures used to examine IAMs pertain to the natural context of experiencing IAMs. Researchers are interested in designing experimental strategies that are the closest approximation to experiencing IAMs in everyday life, in order to thoroughly and exhaustively examine the basic mechanisms of IAMs. Though IAMs, by definition, come to mind without any preceding conscious attempt at retrieval [1, 2], it can be argued that instructing participants in the laboratory to monitor their stream of awareness only for memories may change the nature of involuntary retrieval. For example, participants who know the true goal of a study may retrieve memories voluntarily in order to please the experimenter. Another possibility, which is not mutually exclusive, is that instructing participants to record only spontaneous memories can, *per se*, induce their occurrence. These potential problems may undermine the ability to analyse IAMs as they naturally occur in real life.

A recently published study by Vannucci et al. [29] addressed this issue. The two groups of participants in that study were instructed either to record only IAMs (*the restricted group*) or to record any involuntary thoughts (*the unrestricted group*). Importantly, the word “memory” was completely avoided in the unrestricted group. By manipulating the type of instructions (the restricted vs. the unrestricted condition), Vannucci et al. provided evidence that making participants aware of the goal of the study changed the frequency of IAMs and partially changed their phenomenological characteristics. Briefly, they observed more memories in the restricted group, whose memories also were more specific and had been rehearsed more often in the past, compared with the memories of the unrestricted group.

Vannucci et al. [29] proposed several possible effects of instructions that focus on involuntary memories, which may be responsible for the observed differences. The explanations are not mutually exclusive, and thus, the proposed effects may operate simultaneously. First, monitoring the flow of consciousness with the real goal of the study in mind, may activate voluntary retrieval processes **(***inducing voluntary retrieval***)**. Although this is not a deliberate effect, there is also the possibility that participants who are aware of the goal of the study may try to deliberately recall autobiographical memories. Second, instructing participants to record memories may produce some form of selection during retrieval. This may take the form of either placing the focus of attention on retrieval of autobiographical memories or a reporting bias toward memories congruent with what people think involuntary memories should be **(***report bias***)**. The report bias could make participants more prone to report specific memories, i.e. ‘memories that happened at a particular place and time and lasted for a day or less’ ([36], p. 2), and memories related to personal and unusual events. For instance, participants may be more willing to report *having lunch with friends yesterday* rather than *having lunch with a seminar group on Mondays* because the former memory may be perceived as more ‘genuine’, compared to more the general and schematic latter memory.

Lending support to this possible effect, Vannucci et al. [29] found that specific memories were more frequent in the restricted group compared with the unrestricted group. As autobiographical memories are naively considered to refer to special kinds of events, one would also expect unusual and personal events to be reported more often in the restricted group.

Third, there is the possibility that monitoring the stream of consciousness induces a priming effect that makes autobiographical content more available (*priming effect*). For that reason, it may increase the number of memories, in general, and the number of previously activated (e.g. more often rehearsed) memories, in particular. Indeed, Vannucci et al. [29] found, as already mentioned, that the memories reported in the restricted group had been rehearsed more often in the past.

Elaborating further on the third explanation, it may be that focusing on a wide variety of mental content in the unrestricted procedure imposes additional requirements on memories, which they must satisfy to reach awareness. Otherwise memories would not be successfully extracted from the continuous stream of thoughts. As long as participants in the unrestricted group are monitoring the stream of consciousness for unspecified thoughts, the awareness threshold for memories may be set at a relatively high level. For example, less clear and vivid memories would be less accessible compared to memories that are less vague [37–38]. Thus, memories that are more distinctive in terms of their phenomenological characteristics (i.e. more clear, vivid, detailed, or accompanied by stronger emotional and physiological reactions) should be more likely to pass the threshold of consciousness, compared to less distinctive memories that may not be easily noticed among a wide variety of different mental contents. For that reason, memories with less distinctive phenomenological characteristics should be observed more frequently in the restricted group, since those participants would be actively monitoring the flow of mental content only for memories. In other words, looking for memories may boost the likelihood that they pass the awareness threshold (*threshold effect*).

Whatever processes were actually induced in the participants who were aware of the aim of the Vannucci et al. study [29], the study’s results clearly demonstrated that the type of procedure used can affect the retrieval of IAMs. Equally important, the results highlight the possibility that previous findings on IAMs may be related only to some types of IAMs. For that reason, there is a need for further research to replicate Vannucci et al.’s findings and to verify the explanations for the instruction effects that they advanced.

### 1.2. The present study

The overall goal of the present study was to address an important methodological question; namely, whether the experimental instructions commonly used in studies on IAMs change both the characteristics and frequency of IAMs. This goal is related to the broader issue of the validity of the experimental procedures used in IAMs research. Therefore, the first aim of the present study was to replicate the 2014 study by Vannucci et al. [29], while overcoming some of its limitations. As the authors themselves pointed out, a major limitation of the study was its relatively small sample (12 participants in the restricted group and 11 in the unrestricted group). We used basically the same experimental method of eliciting IAMs [19] as in the Vannucci et al study, and our sample was more than twice as large. They also avoided the word ‘memory’ in the instructions given to the unrestricted group, as not mentioning memory could lead to a selection bias during retrieval by increasing the risk of not noticing some of the IAMs. In other words, participants might be more prone to omit some of their memories since they were monitoring the stream of consciousness for an unspecified category of thoughts. We could not be sure if they would treat autobiographical memories as a part of the vague category of involuntary thoughts. Hence, a smaller number of memories and a lower proportion of specific memories observed in the unrestricted group might occur due to selection bias towards a naively understood concept of ‘thoughts’ during retrieval. To avoid this potential effect, we provided both the restricted and unrestricted group with examples of thoughts (e.g. personal goals, words, current concerns, plans, and memories) when we gave the instructions. Thus, the unrestricted group was not instructed to report memories, but memories were mentioned when explaining the concept of involuntary thoughts.

We hypothesised that, despite some differences in methodology, we would replicate the main finding of Vannucci et al. [29] that involuntary memories were reported more often in the restricted group compared to the unrestricted group.

The second aim of our study was to test three mechanisms that may explain the effect of instructions on the number and characteristics of IAMs. To this end, we compared IAMs reported by the unrestricted and restricted groups on characteristics that have not been previously studied. We employed a self-report scale of the effort that participants put into retrieving memories and thoughts to test the possibility that instructions focusing on memories may induce voluntary retrieval processes. The scale enabled us to analyse the extent to which memories and thoughts were initiated in a goal-oriented fashion. If the restricted procedure enhances voluntary retrieval we should observe higher ratings on the memory effort scale in the restricted group, compared to the unrestricted group.

To test the threshold effect explanation, which presumes that looking for memories boosts the likelihood that they will pass the awareness threshold, we compared various phenomenological characteristics of the memories. We expected that the restricted group would report memories with less distinctive characteristics (i.e. less clear, vivid, and detailed, and less likely to evoke emotional and physiological reactions) compared to the unrestricted group. We asked participants to rate the phenomenological properties of their involuntary thoughts or memories as soon as they occurred during the vigilance task. We decided to have the memories rated during the vigilance task because we wanted them to be rated as they were experienced when they were (presumably) retrieved involuntary, rather than after the vigilance task, when they were intentionally retrieved to complete the post-task assessment. The phenomenological characteristics of a memory that has been involuntary retrieved may be slightly different from the characteristics of a memory of the same event that was intentionally recalled later [39]. One inevitable disadvantage of ongoing (i.e. during the vigilance task) ratings is they may interfere with the process of eliciting and reporting IAMs (see Vannucci [29] for arguments against ongoing ratings). We kept the ongoing ratings brief to minimise their interference with the main retrieval task.

To test the claim that instructions that focus on memories lead to a bias toward reporting memories congruent with what people think involuntary memories should be, we compared the events to which the memories referred in terms of how specific, unusual, and personal they were. We expected that events described by the restricted group would be rated higher on these dimensions.

## Method

This study was approved by the Research Ethics Committee of the Institute of Psychology at Jagiellonian University. Written consent for participation was obtained prior to data collection.

### Participants

Sixty undergraduate students (age range = 19–38 years, *M*_age_ = 22.41; *SD* = 3,06, Female = 41) participated in this study in return for a gift-card worth about $7. They were randomly assigned to one of two conditions (We assigned participants into conditions using simple randomization, i.e. flipping a coin [40]): instructions that did or did not tell participants to record only IAMs. All participants were screened for depression using the Polish version of the Beck Depression Inventory [41]. Four participants who scored 20 or above were excluded from further analysis. Due to technical difficulties, one person did not finish the experiment. The final number of participants was as follows: (a) the restricted group (i.e. participants recording only IAMs—age range = 19 to 29 years, *M*_age_ = 22.27; *SD* = 2.51, Female = 21, Male = 8); and (b) the unrestricted group (i.e. participants recording any involuntary thoughts—age range = 22 to 28 years, *M*_age_ = 22.06; *SD* = 1.94, Female = 20, Male = 6).

### Materials

#### Vigilance Task

The experiment employed a slightly modified procedure from that originally developed by Schlagman and Kvavilashvili [19]. The Involuntary Memories Program (IMP) was constructed using the C# programming language, Microsoft.NET Framework 4.0, and XNA libraries. Briefly, the IMP simulates the conditions in which IAMs have been observed most frequently in daily life using a naturalistic diary method (e.g. [1]), namely, an automatic activity that did not demand much attention. Participants performed an uninteresting vigilance task that involved detecting a pattern of 15 vertical lines in a stream of 785 horizontal lines, with each set of lines presented for 2 seconds during each separate trial. In addition, short verbal phrases (e.g. riding a bicycle, listening to the radio) were displayed on each trial in the centre of the screen. The participants were informed that involuntary thoughts (the unrestricted group) or involuntary memories (the restricted group) were likely to occur during the task, and they were instructed to record them each time they experienced them. Unless specified otherwise, we strictly followed the original study design (for further details see: Schlagman & Kvavilashvili [19]). The IMP used in the present study differed from Schlagman and Kvavilashvili’s [19] original design in the following ways: (1) we randomly ordered the trials for each participant, (2) we extended the presentation of each trial from 1.5 to 2 seconds, (3) the participants were not to say “yes” aloud when they detected a target stimulus; they had to push a red button instead.

#### Polish adaptation of verbal phrases

A total of 1,057 phrases were prepared, of which 800 came from Schlagman and Kvavilashvili’s study [19]. The latter phrases were translated into Polish by three independent research assistants who were proficient in English. The final versions across translations were determined by using the National Corpus of Polish (http://nkjp.pl/), which is a collection of commonly used words and phrases [42]. Therefore it was possible to choose the most frequent and typical collocation of translated phrases (i.e. those with highest chi-square parameter: According to McEnery, Xiao, and Tono [43], chi-square is the most common statistical test used in corpus linguistics. As they explained (p. 55), ‘the chi-square compares the difference between the observed values (e.g. the actual frequencies extracted from corpora) and the expected values’. The higher the chi-square parameter, the better the collocation is, and the less likely it is that the collocation is due to chance). In addition, a Polish linguist checked the translations for grammar, inflection and phraseology. Finally, all the phrases were rated as neutral, positive, or negative by twelve independent coders (6 females, *M*_age_ = 21.42, *SD* = 2.54, range = 20–27), who performed on average or higher in the Test of Emotional Intelligence [44]. Similar to the original procedure used by Schlagman and Kvavilashvili [19], we used the percentage of agreement on perceived type of phrases as a measure of inter-coder reliability. Phrases with an inter-coder agreement of 66% or above (This means the verbal phrase was categorized as neutral if at least 8 of the 12 coders rated it as neutral) were included in the final pool of 800 phrases, which consisted of approximately equal numbers of neutral (*N* = 267), positive (*N* = 267), and negative (*N* = 266) phrases.

#### Questionnaire

The paper questionnaire consisted of two parts. During the vigilance task, participants rated the phenomenological characteristics of the involuntary mental content they experienced (Part 1). After completing the vigilance task, they answered a few questions about the characteristics of the remembered events to which the memories referred (Part 2). S2 Appendix shows the two parts of the questionnaire.

Participants completed Part 1 immediately after experiencing either a memory (the restricted group) or any involuntary mental content (the unrestricted group). After providing a brief description of the memory or thought, they rated (on 7-point scales): (a) the extent to which they had actively tried to bring the thought to mind (henceforth called the effort scale, 1 = *I wasn’t trying at all*, 2 = *I wasn’t trying*, 3 = *I don’t think that I tried*, 4 = *I tried a little bit*, 5 = *I tried somewhat*, 6 = *I tried*, and 7 = *I tried very hard*) (Please note that the scale can actually be considered a dichotomy around the mid-point (4), where 1–3 refer to lack of effort (i.e. effortlessly retrieved), 4 is undecided, and 5–7 explicitly involve effort (i.e. effortfully retrieved). Thus, this scale reflects participants’ confidence in their introspective judgment of effort. So, as the numbers increase from 1–3, the participants are less confident that no effort was involved, but nonetheless all these points assume that retrieval was effortless to some degree. Higher ratings on this scale indicate that the memories are less involuntary, and therefore, more voluntary); (b) the clarity of the thought (henceforth called clarity: 1 = *not clear at all*, 2 = *unclear*, 3 = *rather unclear*, 4 = *a little bit clear*, 5 = *somewhat clear*, 6 = *clear*, and 7 = *perfectly clear*); (c) its vividness (1 = *not at all vivid*, 2 = *very low vividness*, 3 = *low vividness*, 4 = *slightly* vivid, 5 = *somewhat vivid*, 6 = *vivid*, and 7 = *very vivid*); (d) how detailed the content was (1 = *not detailed at all*, 2 = *very low detail*, 3 = *low detail*, 4 = *slightly detailed*, 5 = *somewhat detailed*, 6 = *detailed*, and 7 = *extremely detailed*); (e) the emotional valence of the content (1 = *very unpleasant*, 2 = *unpleasant*, 3 = *rather unpleasant*, 4 = *neutral*, 5 = *rather pleasant*, 6 = *pleasant*, and 7 = *very pleasant*); (f) intensity of emotions experienced in response to the content (1 = *not intense at all*, 2 = *not intense*, 3 = *rather not intense*, 4 = *slightly intense*, 5 = *somewhat intense*, 6 = *intense*, and 7 = *extremely intense*); and (g) the extent to which content was accompanied by unexpected physiological sensations (henceforth, called physiological sensation, 1 = *not accompanied at all*, 2 = *not accompanied*, 3 = *rather not accompanied*, 4 = *difficult to indicate*, 5 = *somewhat accompanied*, 6 = *accompanied*, and 7 = *extremely accompanied*).

Participants completed Part 2 of the questionnaire after completing the vigilance task. They described their memories more thoroughly and rated (on 7-point scales): (a) the emotional valence of the event to which the memory referred (1 = *very unpleasant*, 2 = *unpleasant*, 3 = *rather unpleasant*, 4 = *neutral*, 5 = *rather pleasant*, 6 = *pleasant*, and 7 = *very pleasant*); (b) how often they had recalled the event in the past (henceforth called rehearsal, 1 = *not recalled at all*, *it was the first time*, 2 = *recalled very rarely*, 3 = *recalled rarely*, 4 = *recalled somewhat often*, 5 = *recalled rather often*, 6 = *recalled often*, and 7 = *recalled a great deal*); (c) how personal it was (1 = *not personal at all*, 2 = *not personal*, 3 = *rather not personal*, 4 = *slightly personal*, 5 = *somewhat personal*, 6 = *personal*, and 7 = *extremely personal*); and (d) how unusual the remembered event was (1 = *not unusual at all*, 2 = *not unusual*, 3 = *rather not unusual*, 4 = *slightly unusual*, 5 = *somewhat unusual*, 6 = *unusual*, and 7 = *extremely unusual*).

In addition, they specified whether the remembered event was general or specific, by classifying the event as extended in time (e.g. last winter), as repeated in the past (e.g. regular meetings), or referring to a particular situation that happened on one day (e.g. the day I met my best friend). The first and the second options were classified as general events, while the third one was classified as a specific event.

### Procedure

#### Design

We used a between-subjects design, with the instruction manipulation (with or without an instruction to record only involuntary memories) as the between factor. The instructions are provided in S1 Appendix.

Participants were tested in groups of 2 to 6. They were informed they were free to withdraw from the study at any point. In addition, the experimenter assured them that their responses would be anonymous, and informed them that they could refrain from reporting particularly sensitive thoughts by typing “X” as an answer, or (if possible) by providing a general description of their thoughts rather than a detailed account.

#### Unrestricted procedure

Participants were instructed to report any spontaneously occurring thoughts. They were informed that during the task they might experience different kinds of thoughts, and they were provided examples of such thoughts, including personal goals, words, current concerns, plans, and memories. However, memories were not given particular emphasis during the briefing. The participants were asked to report any spontaneous thoughts that occurred during the 800 vigilance trials, by pressing the spacebar as soon as they became aware of them. Immediately after pressing the spacebar, they provided a brief description of the content of their thoughts and completed Part 1 of the paper questionnaire. After answering the questions in Part 1, the participants clicked “continue” to return to the vigilance task. After completing the vigilance task, participants answered open-ended questions concerning the true goal of the study. Then, they were given written and verbal information describing the nature of autobiographical memory (as, for example, in [45]) and informed about the second part of the study (see S2 Appendix). During the second part, the participants reviewed all the thoughts they recorded during the vigilance task, and were asked to decide whether each thought was or was not an autobiographical memory. Participants then described their memories more thoroughly and completed Part 2 of the paper questionnaire.

#### Restricted procedure

The only difference between this condition and the unrestricted condition was that participants were instructed to report only IAMs that spontaneously came to mind during the vigilance task.

## Results

Entries that were not coded as autobiographical memories by the participants were excluded from the data analysis. Examples of non-autobiographical entries were plans for the future (*going to the cinema tomorrow*), semantic thoughts (*song lyrics*), episodic memories unrelated to the self (*people gathering at the Place de la Republique to pay homage to the Paris attack victims*), and thoughts about the present situation (*how long will it take me to finish this boring task*). None of participants in the unrestricted condition reported having guessed the real purpose of the study. The overall mean ratings of all the dependent variables are reported in Table 1. Cohen’s *d*’s were calculated as the measure of effect size for every *t-*test result [46].

**Table 1:** The overall means ratings for the characteristics of memories as a function of group and all the comparisons of memory types, with *t*-values, *p*-values, critical *q*-values, and effect sizes.

	Group							
	Restricted procedure		Unrestricted procedure					
	*M*	*SD*	*M*	*SD*	*t*	*p*	*q*	*d*
Individuals’ number of memories	7.55	5.77	4.48	3.23	2.45	0.018	n/a	0.64
Clarity*	4.87	1.66	5.51	1.22	3.95	0.001	0.004	0.42
How detailed it was*	4.27	1.82	4.88	1.56	3.00	0.003	0.008	0.35
Vividness*	4.32	1.84	4.88	1.52	2.94	0.004	0.013	0.32
Unusual	4.40	1.91	3.80	1.72	2.73	0.007	0.017	0.32
Effort*	2.24	1.31	1.92	1.01	2.48	0.014	0.021	0.26
Physiological sensation*	2.86	1.85	3.36	1.74	2.38	0.018	0.025	0.28
Personal nature	4.16	1.96	3.60	2.06	2.38	0.018	0.029	0.28
Intensity of emotions*	4.01	1.72	4.18	1.44	0.92	0.358	0.033	0.15
Rehearsal	3.01	1.60	2.90	1.66	0.61	0.541	0.038	0.07
Valence of the event	4.48	1.92	4.41	1.93	0.33	0.740	0.042	0.04
Specific memories	0.66	0.22	0.65	0.37	0.18	0.886	0.046	0.03
Valence of the memory*	4.61	1.73	4.62	1.78	0.04	0.966	0.050	0.01

Tests above the line are statistically significant at the corrected *q* = .029 level (the corrected *q* applies to the phenomenological characteristics). Online ratings are marked with an asterisk.

### The number of memories

Participants recorded 112 memories (e.g. buying flowers for a girlfriend, a first kiss, celebrating an 18^th^ birthday) in the unrestricted group and 219 in the restricted group. Only one participant in the unrestricted group did not report any IAMs during the vigilance task. The vast majority (85.84%) of all the memories recorded by participants in the restricted condition were reported as effortless retrieval (i.e. memories rated 1 to 3 on the effort scale; n = 188), and effortful retrieval (i.e. memories rated 5 to 7 on the effort scale) was reported for only 16 memories (7.31%). The remaining 15 memories (6.85%) were rated in the middle of the effort scale (i.e. a rating of 4). In the unrestricted condition, effortless retrieval was reported for 104 memories (92.86%) and 2 memories were classified as effortful retrieval memories (1.79%). The remaining 6 memories (5.36%) were rated in the middle of the effort scale. Each participant’s number of IAMs were analysed by a t-test for independent samples to assess the effect of instructions on the number of IAMs. The analysis revealed there was a significant difference in the number of IAMs between the two groups, *t*(45) = 2.45, *p* < .018, *d* = .64 (a large effect size). Participants reported significantly more memories in the restricted group (*M* = 7.55, *SD* = 5.77), in which they were instructed to report only IAMs, than in the unrestricted group (*M* = 4.48, *SD* = 3.23), in which they were instructed to report any spontaneous thoughts.

### Characteristics of the memories

The mean ratings for each memory characteristic were analysed in a series of independent *t-*tests to assess the effect of the experimental manipulation on the phenomenological characteristics of the IAMs. In all, we performed 12 *t*-tests (with unequal variances, when necessary). To control for multiple comparisons, we used the False Discovery Rate correction [47], with *α* = 0.05; the critical value *q* was 0.029. The results of all the tests can be seen in Table 1.

First, we examined how the memories were remembered at the time of their occurrence (recorded online during the vigilance task), and then we considered the characteristics of the remembered events as they were rated when the vigilance task was completed.

#### Phenomenological characteristics recorded online

During the vigilance task, participants rated the extent to which they had actively tried to bring a thought to mind, its vividness and clarity, how detailed the memory was, its emotional valence, its intensity of emotion, and the extent to which the memory’s content was accompanied by physiological sensations. As seen in Table 1, memories in the unrestricted group were rated as significantly more vivid, clear, detailed, and more often accompanied by physiological reactions compared with memories in the restricted group. They also were retrieved with less effort than were memories in the restricted group.

#### Characteristics of remembered events

After completing the vigilance task, participants rated the emotional valence of the event, rehearsal, its personal nature, and how unusual the remembered event was. In addition, they specified whether the memory was general or specific. As seen in Table 1, memories in the restricted group were rated as more unusual and personal compared with memories in the unrestricted group. No other differences were observed between the two groups.

As the mean proportions of specific memories relative to general memories did not differ significantly between the groups, any differences between the memories that were described above were not due to a difference in the specificity of the remembered events.

## Discussion

The main purpose of the present study was to examine the effects of experimental instructions on the frequency and characteristics of IAMs. It addressed the methodological question to what extent experimental procedures that are used to investigate IAMs simulate the naturalistic conditions of experiencing IAMs. Participants were divided into two experimental groups: a group instructed to record only IAMs (the restricted group) and a group instructed to record any spontaneous thoughts (the unrestricted group). This allowed us to manipulate participants’ focus on either memories only or any thoughts that appeared in their consciousness. It is worth noting that both groups were instructed in exactly the same way to report these mental contents as soon as they experienced them.

As expected, and consistent with the 2014 study by Vannucci et al. [29], the frequency of IAMs was higher among the participants instructed to record only IAMs. Consistent with Vannucci et al.’s results, the type of instruction also influenced the phenomenological characteristics of the memories. Thus, we were able to replicate the main findings of Vannucci et al., using a much larger sample and slightly modified procedures that addressed some of the limitations of their study. We did not completely avoid the word “memory” in the instructions given to the unrestricted group, as memory was included as an example of an involuntary thought, so it is less likely that the lower number of IAMs in that group resulted from a bias toward not reporting memories.

Because we compared the reported memories of the restricted and unrestricted groups in terms of dimensions on which they have not been compared before, our study sheds new light on the mechanisms that underlie the effects of instruction on the number and the properties of IAMs.

First and foremost, our results support Vannucci et al.’s claim [29] that instructing participants to record only IAMs makes them more likely to engage, to some extent, in the voluntary retrieval of autobiographical memories. The restricted group had significantly higher ratings on the effort scale compared to the unrestricted group, which indicates that the groups differed in terms of how much participants were trying to bring memories to mind. However, this is not to say that the memories in the restricted group were retrieved in a voluntary fashion. While some of them might have not been fully spontaneous (c.a. 7% of them were rated 5–7 on the effort scale), the overall low ratings on the effort scale (*M* = 2.24, *SD* = 1.31) suggest that the instruction to report only IAMs did not make participants more inclined to deliberately recall past events. It is important to mention that the means observed in both groups were below 4 on the effort scale and a rating of 4 indicated ‘*I was trying a little bit*’. Therefore, the results suggest that the instructions focusing on involuntary memories tended to enhance the extent to which participants accidently retrieved IAMs in a goal-oriented fashion (see Vannucci et al., [29], for a similar argument). The instructions given in the restricted condition seem to have made the process of IAM retrieval more effortful, and thus, less automatic than the instructions in the unrestricted condition. Hence, there was more of an unintentional effort than intentional or deliberate effort to retrieve memories in the restricted group as a result of the instructions.

Second, our results lend support to the notion that instructing participants to record only memories may lower the awareness threshold that is required to report a memory. We found that the unrestricted group rated memories as being more clear, vivid, and detailed, and more likely to be accompanied by physiological reactions. It is reasonable to argue that a memory needs to be somewhat distinctive and accompanied by some sensations for it to capture a participant’s attention and be extracted from the flux of mental content. We argue that one of the main mechanisms underlying the higher frequency of IAMs in the restricted group was the participants’ ability to thoroughly monitor their stream of consciousness and extract autobiographical content from the stream, despite the fact that the content was neither especially distinctive nor arousing. Nevertheless, as participants in the restricted group did not have to spend time writing down any thoughts that came to mind, they may simply have had more time to retrieve, for example, less detailed memories. However, if this was the case, we would expect to observe more specific memories in the restricted group, since participants would have more time to retrieve memories that were less general or abstract. Yet, there was no group difference in the specificity of the memories. In addition, if time had an effect on the reported memories of the participants in the restricted group, one would expect that their memories would be longer elaborate. Hence, the memories in the restricted group should have been more detailed and elaborate than the memories in the unrestricted group, but the observed difference was in the opposite direction.

We did not find support for the idea that the procedure used in the restricted group induced a priming effect that made previously activated autobiographical memories more available. Vannucci et al. [29] suggested that looking only for memories may prime attention to particular autobiographical content and enhance their overall activation. This may help memories to pass the awareness threshold. It is important to recognise that this priming effect should manifest itself mainly by an increase in memories that are already highly accessible and, because of this, easy to retrieve. Thus, one would expect to observe more memories that have been rehearsed frequently in the condition that enhances autobiographical memory retrieval, compared to the condition that does not strongly favour this type of retrieval. Since participants in the restricted condition were specifically focused on memories, we would expect them to report a higher frequency of previously recalled or rehearsed memories compared to the memories reported by the participants in unrestricted condition, as observed in the Vannucci et al. study [29]. Although the memories in the restricted group were numerically rated as being rehearsed more often in the past, compared to the memories of the unrestricted group, the difference was not statistically significant.

Finally, we found group differences in some characteristics of the events to which the memories referred; these characteristics were rated by participants during the post-task phase. This finding may imply that the different retrieval strategies that are induced by the two types of instructions favour certain types of remembered events. For example, participants in the restricted group reported more unusual and personal events compared to the unrestricted group. These results accord with Vannucci et al.’s proposal [29] that participants in the restricted group may be inclined to limit their reports to what they naively think IAMs should be (memories referring to personal and more unusual events). On the other hand, it may be argued that some IAMs in the unrestricted group were episodic memories unrelated to the self. While this might be an issue, it is worth noting that we excluded participant entries from data analysis that were not coded as autobiographical memories. Also, the written and verbal instructions describing the nature of autobiographical memory were identical in both groups (see S2 Appendix). Participants from both groups were also uniformly instructed (see S1 Appendix) to write down any kind of memory (restricted group) or thought (unrestricted group) that arose during the vigilance task, regardless of what it was, or how interesting they found it to be. By doing so, we tried to avoid the participants’ use of a layperson’s definition of autobiographical memory and to reduce the extent to which they might voluntarily limit their responses only to some types of memories or thoughts (for example, those that were more or less unusual). Furthermore, the systematic reporting of more episodic memories unrelated to the self in the unrestricted group should differentiate the groups on the specificity ratio. However, we did not replicate the differences between the groups in the specificity of remembered events.

We argue therefore, that given the identical instructions about autobiographical memory provided to both groups, as well as the lack of differences between the groups in the specificity of remembered events, there is no reason to believe that the groups differed in their general understanding of what autobiographical memory is. At the same time, we still observed differences between the groups in the phenomenological characteristics of the recorded memories. Therefore, we believe that these differences may be better explained by reference to the experimental manipulation, rather than to differences in participants’ understanding of what autobiographical memory is. Finally, it is worth noting that the lack of group differences in the specificity of remembered events suggests that the group differences we found in the phenomenological characteristics of the recorded memories were not likely to be due to differences between specific and general memories.

### Possible limitations

Some limitations should be taken into account when considering the present results. First trying to report every involuntary thought during the vigilance task may have interfered with the natural flow of thoughts that takes place during everyday mind-wandering. Second, reporting every thought that came to mind may have increased the risk that after a certain point the participants stopped reporting any mental content because the task became too long and onerous. Vannucci et al. [29] considered the second issue to be especially problematic when participants were asked to rate every spontaneous thought during the vigilance task, i.e. as soon as they realized that they had one. They asked participants to do all the ratings after the vigilance task had been completed. To address the possible effect of the task’s burden on the number of IAMs reported, we conducted an exploratory analysis by comparing the mean number of all memories recorded in: (1) the unrestricted group in our study (*M* = 4.48, *SD* = 3.23) vs. the unrestricted group in Vannucci et al.’s study (self-interrupted: *M* = 6.92, *SD* = 6.63), and (2) the restricted group in our study (*M* = 7.55, *SD* = 5.77) vs. the restricted group in Vannucci et al.’ study (self-interrupted: *M* = 9.25, *SD* = 6.17). A two-tailed *t*-test did not find a statistically significant difference between the studies either in the former comparison (*p* = 0.14) or in the latter comparison (*p* = 0.41). Although the analysis is only exploratory, it suggests that the online rating procedure did not significantly decrease the likelihood of reporting memories/thoughts by participants throughout the task. However, this issue still needs to be addressed in future studies.

### Final conclusions

In accordance with Vannucci et al.’s [29] findings, our results indicate that instructing participants to record only IAMs significantly increases the frequency of experiencing IAMs. It is highly likely that participants in the restricted group may have been more willing to notice past-related thoughts in their stream of consciousness or they may have been more willing to focus on them compared to the unrestricted group. The pattern of differences in phenomenological characteristics between memories reported in the restricted and unrestricted groups lends credence to this claim. Therefore, our study supports the idea that the different research procedures that were used in previous experiments on IAMs might have affected the way in which involuntary memories were retrieved. Importantly, different retrieval strategies may also favour certain types of memories.

According to Michael, Garry, and Kirsch [48], expectations of a particular outcome, (e.g. the expectation of experiencing IAMs or involuntary thoughts) may automatically modify our cognition and behaviour to produce that outcome. Thus, the ubiquitous results of the expectancy effect may be responsible for well-known effects in psychological science. The involuntary memory research field may be especially vulnerable to this kind of phenomenon, and therefore, one should take this into consideration when designing experimental procedures.

## Supporting Information

S1 Appendix Instructions received by all the participants. (PDF)

S2 Appendix Paper questionnaire (Part 1 and 2) received by all the participants. (PDF)
