# Supplementary material for: The Effects of Instruction on the Frequency and Characteristics of Involuntary Autobiographical Memories
Source: PLoS One. 2016 Jun 13;11(6):e0157121. doi: 10.1371/journal.pone.0157121 (PMC4905669; doi:10.1371/journal.pone.0157121)
Supplement: S1 Appendix — (PDF) [file pone.0157121.s001.pdf]

Appendix S1

The goal of this task is to study people's ability to concentrate long on relatively monotonous and repetitive stimuli. You will be shown a large number of different patterns of either horizontal or vertical lines on a computer screen. Each time you see vertical lines on the screen please press the red button on the keyboard. Apart from the lines there will be also some word phrases displayed in the center of the screen. The condition you are taking part in addresses the ability to concentrate on relatively monotonous stimuli regardless of the fact that words and phrases are displayed at the same time. In another condition, participants have to concentrate on the words and phrases regardless of the fact that patterns of lines are displayed at the same time. For your better understanding of the task you are about to perform a short practice trial. Remember, each time you will see vertical lines press the red button on the keyboard.

[After completing the trial session]

As you know, this task is to study people's ability to concentrate long on relatively monotonous stimuli. For that reason, during the task you may find yourself unintentionally thinking about other things, which is quite normal. Information about thoughts that you may experience during the task is very important for us. It will allow us to understand peoples' ability to concentrate on monotonous stimuli.

Unrestricted condition. For that reason, each time you find yourself thinking about other things press the spacebar as soon as you become aware of it. It will stop the program and allow you to record the thought on the paper sheet that you will find on the table. Note that these thoughts may concern different things – for example, simple associations, words and facts. They may also be more elaborated and concern current projects, future goals, or memories related to something from your past, i.e. something you have witnessed or experienced. No matter what type of spontaneously occurring thought you experience, please report it as soon as you become aware of it by pressing the spacebar. It is not important what it will be and how interesting you will find your thought. Just press the spacebar each time you have any spontaneously occurring thought and fill in the paper sheet. You can refrain from reporting particularly sensitive thoughts by typing "X" as an answer or (if possible) by providing a general description of your thought rather than a detailed account.

Restricted condition. Note that these thoughts may concern different things – for example, simple associations, words and facts. They may also be more elaborated and concern current projects, future goals, or memories related to something from your past, i.e. something you have witnessed or experienced. Each time you find yourself experiencing a spontaneously occurring memory please press the spacebar as soon as you become aware of it. It will stop the program and allow you to record the memory on the paper sheet that you will find on the table. No matter what type of spontaneously occurring memory you experience, please report it as soon as you become aware of it by pressing the spacebar. It is not important what it will be and how interesting you will find your memory. Just press the spacebar each time you have any spontaneously occurring memory and fill in the paper sheet. You can refrain from reporting particularly sensitive memories by typing "X" as an answer or (if possible) by providing a general description of your memory rather than a detailed account.
