# Supplementary material for: The Effects of Instruction on the Frequency and Characteristics of Involuntary Autobiographical Memories
Source: PLoS One. 2016 Jun 13;11(6):e0157121. doi: 10.1371/journal.pone.0157121 (PMC4905669; doi:10.1371/journal.pone.0157121)
Supplement: S2 Appendix — (PDF) [file pone.0157121.s002.pdf]

Questionnaire – Part I

Please write down briefly the content of your thought.
It is not important what it is and how interesting you find it.
Just describe it in a few words the same way you have just experienced it.
You can refrain from reporting particularly sensitive contents by typing "X" as an answer
or (if possible) by providing a general description of your thought rather than a detailed account.

....

1.	I wasn't trying at all	I wasn't trying	I don't think that I tried	I tried a little bit	I tried somewhat	I tried	I tried very hard
Did you deliberately try to bring the content to mind?	1	2	3	4	5	6	7

2.	Not at all accompanied	Not accompanied	Slightly not accompanied	Difficult to indicate	Somewhat accompanied	Accompanied	Extremely accompanied
Indicate the extent to which the content was accompanied by any kind of unexpected physiological sensations (e.g. increased heart rate, heat feeling inside your body, sweating and so on):	1	2	3	4	5	6	7

3.

	Not clear at all	Unclear	Rather unclear	A little bit clear	Somewhat clear	Clear	Perfectly clear
How clear do you remember the content of this thought:	1	2	3	4	5	6	7

4.

	Not at all vivid	Very low vividness	Low vividness	Slightly vivid	Somewhat vivid	Vivid	Extremely vivid
How vivid is this content:	1	2	3	4	5	6	7

5.

	Not detailed at all	Very low detail	Low detail	Slightly detailed	Somewhat detailed	Detailed	Extremely detailed
How detailed is this content:	1	2	3	4	5	6	7

6.

	Very unpleasant	Unpleasant	Rather unpleasant	Neutral	Rather pleasant	Pleasant	Very pleasant
This content is for you:	1	2	3	4	5	6	7

7.

	Not intense at all	Not intense	Rather not intense	Slightly intense	Somewhat intense	Intense	Extremely intense
How intense are emotions experienced in response to this content:	1	2	3	4	5	6	7

If you have answered all the questions – please go back to the programme

Questionnaire – part II

The following questions relate to the content you wrote down during the first part of the present study.

If this content is a memory and relates to something from your personal past, i.e. something that you personally experienced, saw or witnessed – please answer the following questions. Memory may be of an event, situation, or experience from your past. It is not important what it is and how interesting you find this memory.

Please note that this memory may relate to something from your recent or remote past. It may describe an event that was: (1) more general and extended in time (e.g. last winter), or (2) repeated in the past (e.g. attending math class last year) or (3) specific and referring to a particular situation that happened at a particular place and time and lasted for a day or less (e.g. unexpected meeting with a relative last Monday).

If this content was something that does not relate to anything from your past – go to the next thought you wrote down.

All following questions relate to the memory you described in the first part of this questionnaire.

2. Please describe the memory more thoroughly:

....

....

3.	More general, extended in time, lasted some time (e.g. last summer)	An event that was repeated in the past (e.g. regular meetings)	Referring to a particular situation happening within one day (e.g. passing driving test)
The memory may be described as:	1	2	3

4.	Very unpleasant	Unpleasant	Rather unpleasant	Neutral	Rather pleasant	Pleasant	Very pleasant
The original event was for you:	1	2	3	4	5	6	7

5.	Not recalled at all, it was the first time	Recalled very rarely	Recalled rarely	Recalled somewhat often	Recalled rather often	Recalled often	Recalled a great deal
How often have you recalled this memory in the past:	1	2	3	4	5	6	7

6.	Not personal at all	Not personal	Rather not personal	Slightly personal	Somewhat personal	Personal	Extremely personal
How personal was this event:	1	2	3	4	5	6	7

7.	Not unusual at all	Not unusual	Rather not unusual	Slightly unusual	Somewhat unusual	Unusual	Extremely unusual
How unusual the remembered event was:	1	2	3	4	5	6	7

If you have answered all the questions – please go to the next questionnaire
